# Inhibitors of AKT kinase increase *LDL receptor* mRNA expression by two different mechanisms

**DOI:** 10.1371/journal.pone.0218537

**Published:** 2019-06-19

**Authors:** Katrine Bjune, Lene Wierød, Soheil Naderi

**Affiliations:** Unit for Cardiac and Cardiovascular Genetics, Department of Medical Genetics, Oslo University Hospital, Oslo, Norway; North Carolina State University, UNITED STATES

## Abstract

Protein kinase B (AKT) is a serine/threonine kinase that functions as an important downstream effector of phosphoinositide 3-kinase. We have recently shown that MK-2206 and triciribine, two highly selective AKT inhibitors increase the level of *low density lipoprotein receptor (LDLR)* mRNA which leads to increased amount of cell-surface LDLRs. However, whereas MK-2206 induces transcription of the LDLR gene, triciribine stabilizes *LDLR* mRNA, raising the possibility that the two inhibitors may actually affect other kinases than AKT. In this study, we aimed to ascertain the role of AKT in regulation of *LDLR* mRNA expression by examining the effect of five additional AKT inhibitors on *LDLR* mRNA levels. Here we show that in cultured HepG2 cells, AKT inhibitors ARQ-092, AKT inhibitor VIII, perifosine, AT7867 and CCT128930 increase *LDLR* mRNA levels by inducing the activity of LDLR promoter. CCT128930 also increased the stability of *LDLR* mRNA. To study the role of AKT isoforms on *LDLR* mRNA levels, we examined the effect of siRNA-mediated knockdown of AKT1 or AKT2 on LDLR promoter activity and *LDLR* mRNA stability. Whereas knockdown of either AKT1 or AKT2 led to upregulation of LDLR promoter activity, only knockdown of AKT2 had a stabilizing effect on *LDLR* mRNA. Taken together, these results provide strong evidence for involvement of AKT in regulation of *LDLR* mRNA expression, and point towards the AKT isoform specificity for upregulation of *LDLR* mRNA expression.

## Introduction

Low density lipoprotein (LDL) is the major cholesterol-carrying lipoprotein in humans, making up approximately 70% of circulating cholesterol [1]. The maintenance of plasma LDL-cholesterol is primarily carried out by LDL receptor (LDLR) that mediates the endocytotic clearance of LDL from plasma [1]. Thus, mutations in genes that cause a reduction in LDLR levels or function lead to elevated plasma LDL-cholesterol levels which are associated with an increased risk of cardiovascular disease [1, 2]. Consistent with the essential role of the LDLR in regulation of plasma LDL-cholesterol levels, therapies aimed at increasing LDLR levels, such as statins or antibodies against proprotein convertase subtilisin/kexin type 9, have proven to be the most effective treatments to reduce the incidence of cardiovascular disease [3].

Expression of LDLR is tightly regulated at multiple levels to ensure normal cellular function. At the transcriptional level, the expression of LDLR gene is mainly regulated by sterol regulatory element-binding protein-2 (SREBP-2) [4]. SREBP-2 is a transcription factor that is synthesized as an inactive endoplasmic reticulum (ER) membrane-bound precursor. Upon reduction of intracellular cholesterol levels, SREBP-2 is escorted by SREBP cleavage-activating protein (SCAP) to the Golgi apparatus where SREBP-2 undergoes two sequential proteolytic cleavages to release the transactivation-competent NH2-terminal domain [5, 6]. Once inside the nucleus, the active SREBP-2 binds to its cognate sterol regulatory element-1 (SRE-1) in the *LDLR* promoter and induces *LDLR* expression [7, 8]. However, the amount of *LDLR* mRNA is also regulated by factors that affect the stability of *LDLR* mRNA. *LDLR* mRNA is relatively labile, with a half-life of approximately 2 hours [9], and the stability of *LDLR* mRNA is dictated by regulatory sequences in the 3’untranslated region (3’UTR) that serve as binding sites for mRNA stabilizing and destabilizing trans-regulatory proteins [10, 11].

Even though cholesterol and oxysterol derivatives are the key regulators of SREBP-2, recent studies have shown that SREBP-2 is affected by multiple signaling pathways. One of these pathways is the phosphatidylinositol-3-kinase/protein kinase B (PI3K/AKT) pathway [12]. AKT is a serine/threonine kinase that functions as an important downstream effector of PI3K [12]. In this capacity, AKT integrates and relays signals from a diverse set of extracellular cues to regulate cellular processes such as metabolism, proliferation, growth and survival. Mammalian cells express three structurally homologous AKT isoforms (AKT1, AKT2 and AKT3) that contain an N-terminal pleckstrin homology (PH) domain followed by a central catalytic domain and a C-terminal regulatory domain [13, 14]. The initiating event for activation of AKT is the receptor-stimulated generation of phosphatidylinositol (3,4,5)-triphosphate (PIP3) by PI3K. PIP3 then interacts with the PH domain of AKT and docks it to the plasma membrane where it is activated as a result of phosphorylation on Thr308 in the activation loop and also on Ser473 in the C-terminal regulatory domain by 3-phosphoinositide-dependent protein kinase-1 (PDK1) and mammalian target of rapamycin complex 2 (mTORC2), respectively [15].

We have recently shown that two pharmacologic inhibitors of AKT, MK-2206 and triciribine, increase the levels of *LDLR* mRNA, leading to increased levels of cell-surface LDLRs [16, 17]. Interestingly, we found that MK-2206 and triciribine utilize two different regulatory mechanisms to trigger the accumulation of *LDLR* mRNA. Whereas MK-2206 stimulates *LDLR* gene expression by inducing the proteolytic activation of SREBP-2, triciribine increases the stability of *LDLR* mRNA. This lack of congruence between the two inhibitors that target the same kinase raised the possibility that the divergent effects of MK-2206 and triciribine on LDLR expression might arise from the result of their interaction with targets other than AKT. If this were the case, then it would be highly unlikely that inhibition of AKT by other means would affect the expression of *LDLR*. We addressed this notion by examining the effect of a number of functionally different AKT inhibitors or siRNA-mediated AKT knockdown on *LDLR* expression.

## Materials and methods

### Reagents and antibodies

MK-2206 2HCl, triciribine, AT7867, ARQ-092 and CCT1298930 were obtained from Selleckchem (Houston, TX). AKT inhibitor VIII and perifosine were from AdooQ Bioscience (Irvine, CA). The kinase inhibitors were dissolved in dimethyl sulfoxide (DMSO; Sigma Aldrich, St. Louis, MO), except from perifosine which was dissolved in ethanol. Actinomycin D (ActD) and dithiothreitol (DTT) were from Sigma-Aldrich. Antibodies against LDLR (3839-100) and *β*-tubulin (T9154-05G) were purchased from BioVision (Milpitas, CA) and Nordic BioSite AB (Täby, Sweden), respectively. Antibodies against AKT1 (2938) and AKT2 (2964) were obtained from Cell Signaling (Danvers, MA). siRNAs against AKT1 (Hs_AKT1_7 FlexiTube siRNA) and AKT2 (Hs_AKT2_5 FlexiTube siRNA) were obtained from Qiagen (Hilden, Germany).

### Cell cultures

HepG2 cells (European Collection of Cell Cultures, Salisbury, UK), were cultured on collagen-coated culture vessels (BD Biosciences, San Jose, CA) in HyClone Minimum Essential Medium (GE Healthcare Life Sciences, Pittsburg, PA) containing 10% fetal bovine serum (Sigma-Aldrich), 2 mM L-glutamine (Sigma-Aldrich), 50 U/ml penicillin (GE Healthcare Life Sciences), 50 *μ*g/ml streptomycin (GE Healthcare Life Sciences) and non-essential amino acids (Biowest, Nuaillé, France). The cells were grown in monolayer in an atmosphere of 5% CO_2_ at 37°C. CHO T-REx cells (Invitrogen, Carlsbad, CA) were cultured as previously described [18]. All drugs, except perifosine, were added in DMSO with a constant DMSO concentration of 0.1% (v/v). To control for possible DMSO effects, control samples were treated with DMSO alone at final concentrations of 0.1%.

### Western blot analysis

Cells were lysed in Triton X-100 lysis buffer (20 mM Tris [pH 7.5], 100 mM NaCl, 1% Triton X-100, 10 mM EDTA and Complete Protease Inhibitor Cocktail (Roche, Basel, Switzerland). Equal amounts of proteins were separated by 4-20% sodium dodecyl sulfate polyacrylamide gel electrophoresis. After transfer to a polyvinylidene difluoride membrane (Bio-Rad, Hercules, CA), proteins were detected by use of standard immunoblotting procedures. The band intensities were quantified by the use of Chemidoc Touch Imaging System (Bio-Rad).

### Quantitative real-time PCR

Total RNA was extracted from cells using QIAamp RNA Isolation Kit (Qiagen). cDNA was synthesized with the AffinityScript QPCR cDNA Synthesis Kit (Agilent Technologies, Santa Clara, CA). Quantitative real-time PCR (qPCR) was performed using Brilliant III Ultra-Fast QPCR Master Mix (Agilent Technologies) on Mx3005P QPCR system (Agilent Technologies). The assay ids of the PrimeTime Predesigned qPCR Assays used (Integrated DNA Technologies, Coralville, IA) are shown in Table 1. The experiments were carried out in duplicate. The housekeeping gene glyceraldehyde-3-phosphate dehydrogenase (GAPDH) was used for normalizing the amount of target mRNA. Relative mRNA expression was calculated using the 2^−ΔΔ*Ct*^ method [19].

**Table 1:** The assay ids of the PrimeTime Predesigned qPCR Assays used.

Gene symbol	Assay ID	Ref.seq	Exon location	Assay configuration
*AKT*1	Hs.PT.58.26215470	NM_001014431(3)	4-5	Std, FAM/ZEN/IBFQ, P:P 2
*AKT*2	Hs.PT.56a.3591556.g	NM_001243027(3)	6-8	Std, FAM/ZEN/IBFQ, P:P 2
*LDLR*	Hs.PT.58.14599757	NM_000527(6)	8-9	Std, FAM/ZEN/IBFQ, P:P 2
*GAPDH*	Hs.PT.39a.22214836	NM_002046(1)	2-3	Std, FAM/ZEN/IBFQ, P:P 2

### Plasmids, transfection and reporter assay

The luciferase reporter plasmid containing the LDLR promoter sequence +58 to -1563, pLR1563-luc [20], was a gift from Dr. Youngmi Kim Pak (University of Ulsan College of Medicine, Seoul, Republic of Korea). For plasmid transfections, cultured HepG2 cells were transfected with 312 ng plasmid DNA/cm^2^ using FuGENE HD (Promega, Madison, WI) according to the manufacturer’s instructions. A ratio between FuGENE HD and plasmid DNA of 4.5:1 was used. Cells transfected with empty vector were used as a control. AKT inhibitors were added to the cells 24 h after transfection and harvested after 14 h post-addition. Analysis of reporter gene activities was performed by the use of Dual-Luciferase Reporter Assay (Promega), according to the manufacturer’s instructions. For gene knockdown studies, HepG2 cells were transfected with 40 pmol gene-specific or non-targeting (NT) AllStars negative siRNA (Qiagen) using Lipofectamine RNAiMAX (Thermo Fisher Scientific) with cells at 70% confluency. For dual transfection studies with both siRNA and the pLR1563-luc plasmid, cultured HepG2 cells were transfected with 40 pmol siRNA and 312 ng plasmid DNA/cm^2^ with DharmaFECT Duo Transfection reagent (GE Healthcare Life Sciences) at a ratio of 4:1, according to the manufacturer’s instructions. For measurement of LDLR promoter activity, cells were co-transfected with pLR1563-luc and the Renilla luciferase plasmid, phRL (Promega) at a ratio of 9:1. Cells transfected with empty vector and NT AllStars negative siRNA were used as a control.

### RT-PCR analysis of *XBP1* mRNA splicing to identify ER stress

Total RNA was isolated from CHO T-REx cells using QIAamp RNA Blood Mini Kit (Qiagen). One microgram of RNA was reverse-transcribed using Qiagen Onestep RT-PCR kit (Qiagen) and X-Box Binding Protein 1 (*XBP1*) cDNA was synthesized using the 5’ primer: 5’-CACCTGAGCCCCGAGGAG-3’ and the 3’ primer: 5’-TTAGTTCATTAATGGCTTCCAGC-3’. The reverse transcription reaction was run at 50°C for 30 min. PCR cycling conditions included a 15 min polymerase activation step at 95°C, followed by 40 cycles of 1 min denaturation at 94°C, 1 min annealing at 60°C and 1 min extension at 72°C. PCR products were subjected to electrophoresis on a 2% agarose gel at 50 V for 240 min and stained with GelRed Nucleic Acid Gel Stain (Biotium, Inc., Fremont, CA).

### Statistical analyses

All data are expressed as mean ±SD, except data obtained by qPCR which is expressed as mean ±confidential interval. To determine statistical significance an F-test were first conducted to study whether the treated samples had a variance equal to or different from the control. A two-tailed unpaired Student’s t-test was then used for determination of statistical significance. A p-value < 0.05 was considered statistically significant. The *LDLR* mRNA half-life was calculated using least squares regression.

## Results

### AKT inhibitors induce the expression of LDLR

**Fig 1:**
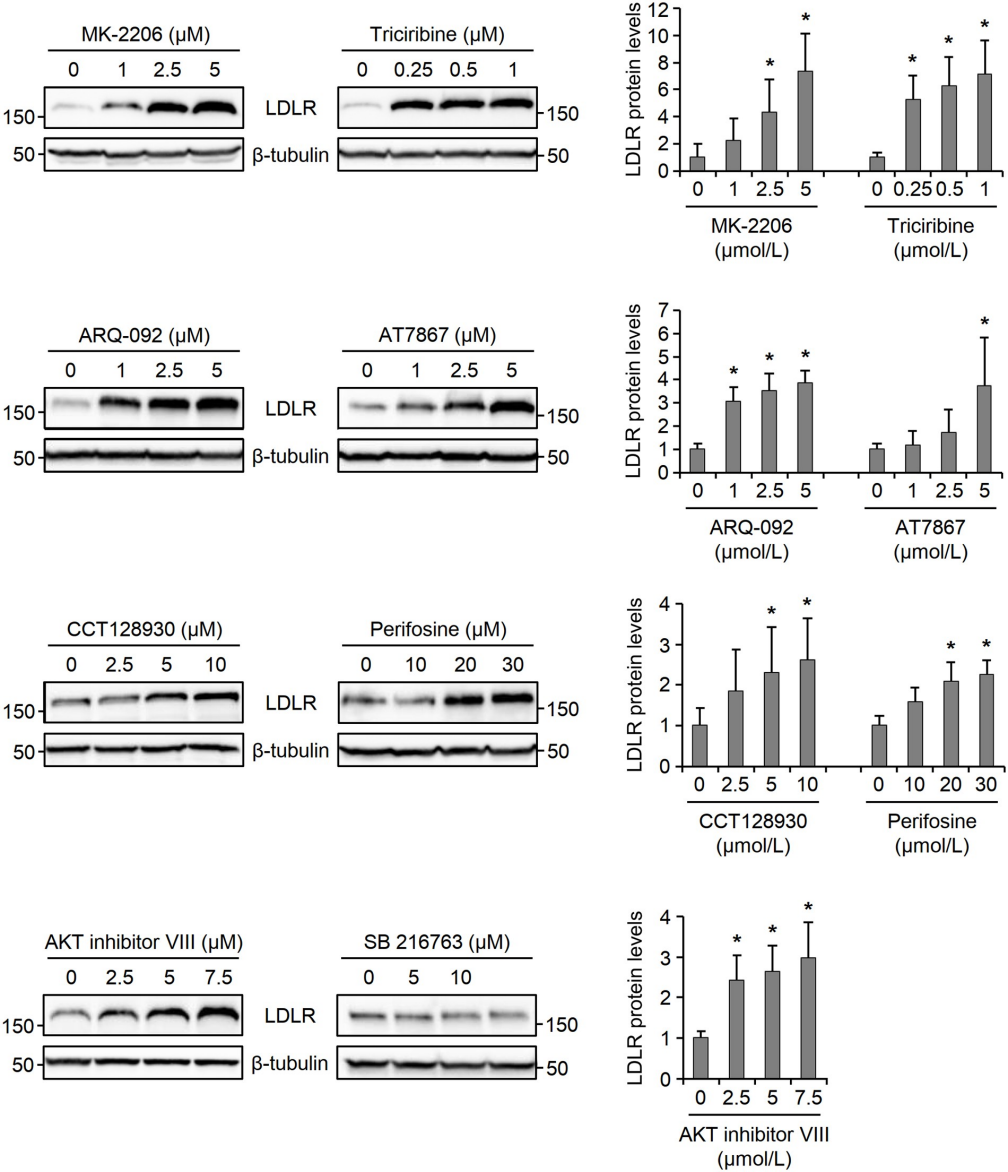
AKT inhibitors increase LDLR levels in a dose-responsive manner. HepG2 cells were cultured in the presence of vehicle, the indicated concentrations of AKT inhibitors or the GSK3*β* inhibitor, SB216763, for 14 h before harvesting for analysis by immunoblotting with antibodies against LDLR and *β*-tubulin. The figure shows one representative blot from four independent experiments. The bar graphs show quantification of the immunoblots which were scanned and the intensity of the LDLR band was normalized to that of *β*-tubulin, and plotted relative to the values obtained for vehicle-treated cells, which were set at 1. Error bars represent SD. *p < 0.5 relative to vehicle-treated cells.

As a first step towards ascertaining whether inhibition of AKT is responsible for induction of LDLR by MK-2206 or triciribine, we examined the effect of additional AKT inhibitors on the LDLR levels. To this end, we set out to examine the expression of LDLR protein levels in HepG2 cells that were exposed to ARQ-092, AKT inhibitor VIII, perifosine, AT7867 or CCT128930 for 14 h. Similar to MK-2206, ARQ-092 and AKT inhibitor VIII are allosteric AKT inhibitors that lock the kinase in a closed, enzymatically inactive conformation [21, 22]. Perifosine is an alkylphospholipid that, similar to triciribine, interferes with the binding of the AKT PH domain to PIP3, consequently inhibiting the kinase activation [23, 24]. AT7867 and CCT138930 are ATP-competitive inhibitors that target and inhibit the phosphorylated conformation of AKT [25, 26]. Except for CCT128930 which exhibits AKT2 isoform selectivity [26], all the above-mentioned inhibitors, including MK-2206 and triciribine, are considered as pan-AKT inhibitors. First, we used phosphorylation of AKT as a readout for its activity to experimentally validate the range of inhibitor concentrations that are reported to inhibit AKT activity [22, 26–30]. Then we treated HepG2 cells with the lowest inhibitor concentration required to markedly inhibit AKT activity, as well as two higher concentrations, and examined the expression of LDLR by Western blot analysis. Fig 1 shows that, similar to MK-2206 and triciribine, all five AKT inhibitors induced the expression of LDLR protein levels in a dose-responsive manner. Importantly, treatment of cells with the GSK3*β*-selective inhibitor, SB216763, did not increase LDLR levels. These data strongly implicate that inhibition of AKT results in induction of LDLR expression.

### AKT inhibitors increase *LDLR* mRNA levels

**Fig 2:**
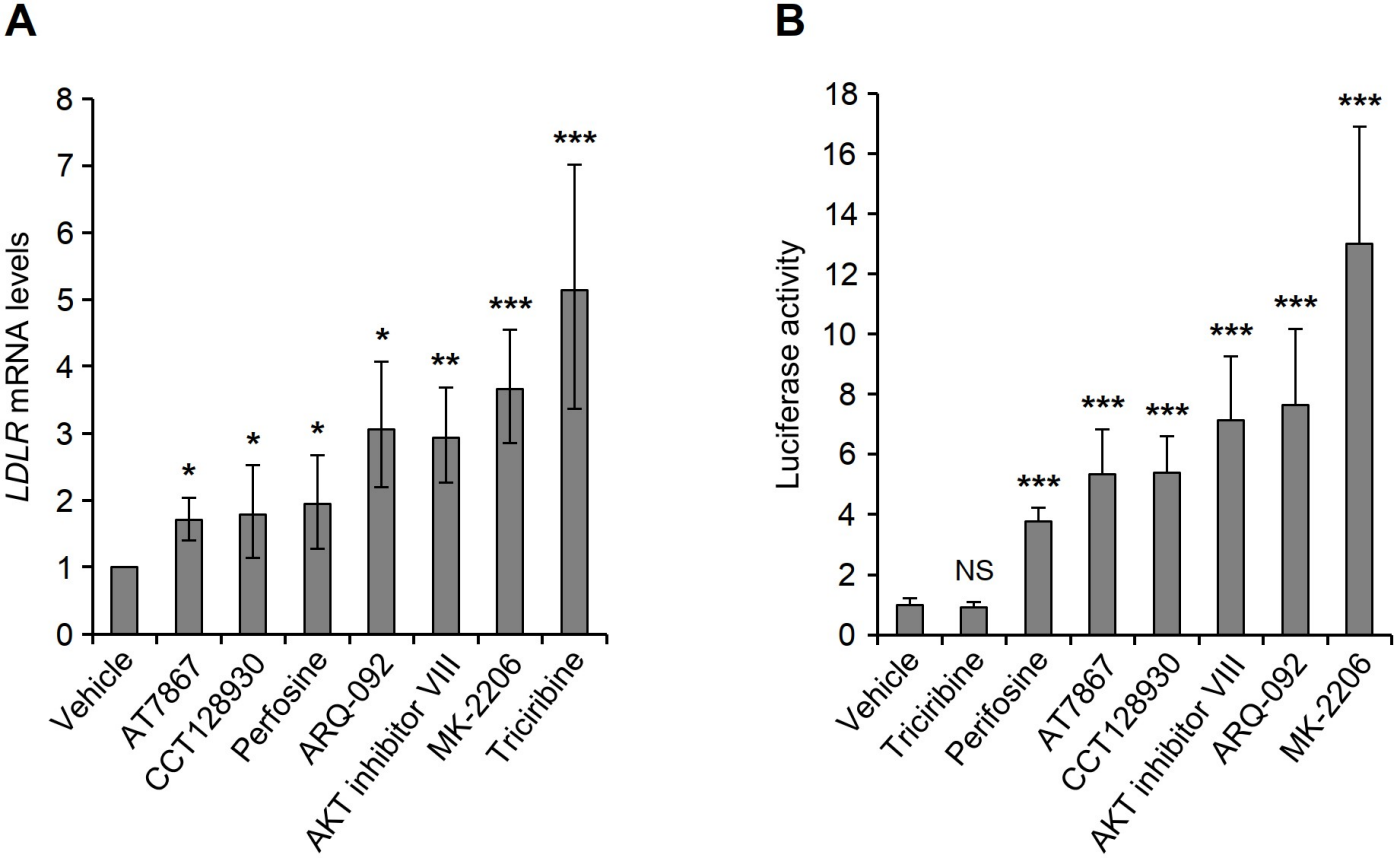
Effect of AKT inhibitors on *LDLR* gene expression and promoter activity. (A) HepG2 cells were treated with vehicle, 5 *μ*M AT7867, 10 *μ*M CCT128939, 30 *μ*M perifosine, 4 *μ*M ARQ-092, 7.5 *μ*M AKT inhibitor VIII, 5 *μ*M MK-2206 or 1 *μ*M triciribine for 14 h before harvesting for isolation of mRNA and determination of *LDLR* and *GAPDH* mRNA levels by qPCR assay. *LDLR* mRNA levels were plotted relative to the values obtained at time 0 (n = 4). (B) HepG2 cells were co-transfected with firefly luciferase reporter pLR1563-luc and Renilla luciferase plasmid. At 24 h post-transfection, cells were treated with vehicle or the indicated AKT inhibitors as in Fig 2A and then harvested for analysis of luciferase activity. After normalization of firefly luciferase activity with Renilla luciferase activity, the averages of data from four independent experiments were plotted relative to the value obtained from vehicle-treated cells that were transfected with pLR1563-luc. Error bars represent the 95% confidence interval. *p < 0.05, **p < 0.01 and ***p < 0.001 compared with vehicle-treated cells.

The MK-2206- and triciribine-mediated accumulation of LDLR occurs as a result of an increase in the *LDLR* mRNA levels. Therefore, we felt it important to examine whether the induction of LDLR by the other AKT inhibitors was associated with an increase in *LDLR* mRNA levels. Quantification of *LDLR* mRNA in HepG2 cells which were treated with AKT inhibitors revealed that, similar to MK-2206 and triciribine, all five inhibitors increased *LDLR* mRNA levels (Fig 2A).

We then proceeded to investigate the mechanism by which the AKT inhibitors increased *LDLR* mRNA levels. To examine the effect of these inhibitors on *LDLR* gene transcription, we cultured HepG2 cells that were transfected with the pLR1563-luc plasmid, a luciferase reporter construct driven by the *LDLR* promoter, in the absence or presence of the AKT inhibitors for 14 h and then examined them for luciferase activity. As expected, luciferase activity was induced in cells that were treated with MK-2206, whereas triciribine had no effect on luciferase activity (Fig 2B) [16, 17]. Interestingly, all the other AKT inhibitors also exerted a positive effect on luciferase activity. These results indicate that, similar to MK-2206, ARQ-092, AKT inhibitor VIII, perifosine, AT7867 and CCT128930 induce transcriptional activity of the *LDLR* gene.

**Fig 3:**
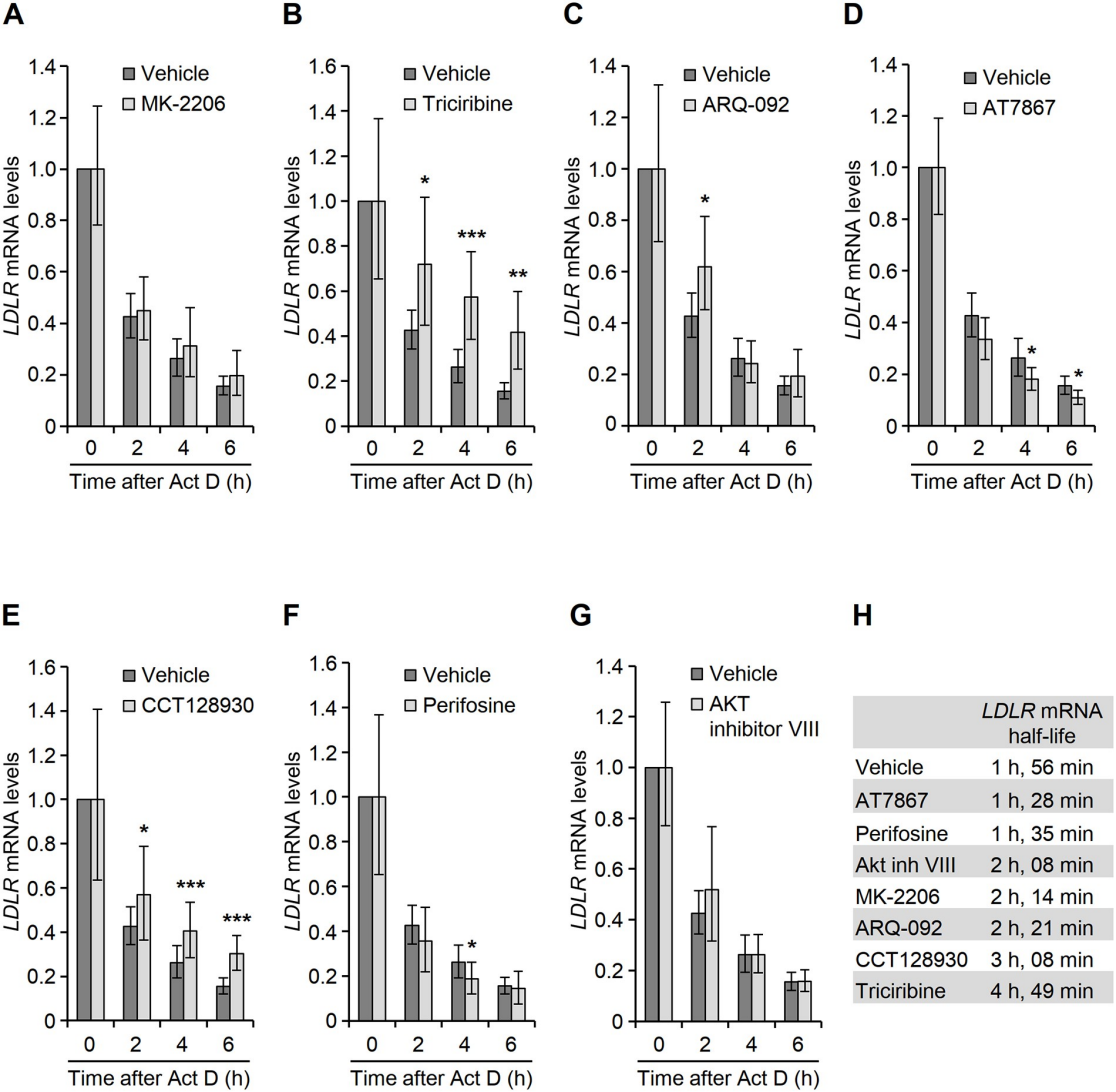
Effect of AKT inhibitors on the stability of *LDLR* mRNA. (A-G) HepG2 cells were treated with vehicle or the indicated AKT inhibitors as described in the legend to Fig 1A before exposure to 5 *μ*g/ml Act D. Cells were then harvested at the indicated time points for determination of *LDLR* and *GAPDH* mRNA levels by qPCR assay. *LDLR* mRNA levels were normalized to those of *GAPDH* and the values were plotted relative to the amount of *LDLR* mRNA before addition of Act D, which was set as 1. The graph shows the mean (±confidential interval) values from five independent experiments. *p < 0.05, **p < 0.01, ***p < 0.001 relative to the amount of *LDLR* mRNA before the addition of Act D. (H) Estimation of the mean lifetime of *LDLR* mRNA in the indicated treatment conditions.

To determine the effect of the AKT inhibitors on *LDLR* mRNA stability, we used the transcriptional inhibitor Act D to examine the effect of the AKT inhibitors on *LDLR* mRNA degradation. Calculation of *LDLR* mRNA half-lives showed that, in accordance with our previous findings, triciribine exerted a potent stabilizing effect on *LDLR* mRNA, whereas MK-2206 minimally inhibited the degradation of *LDLR* mRNA (Fig 3A and 3B). Whereas *LDLR* mRNA exhibited a half-life of approximately 2 h in cells that that were treated with MK-2206, ARQ-092, AT7867, AKT Inhibitor VII or perifosine, it was significantly stabilized in cells that were exposed to CCT128930 or triciribine, with half-life of 3 h or 5 h, respectively (Fig 3A–3H). Together, these data indicate that ARQ-092, AT7867, perifosine, AKT inhibitor VIII and CCT128930 increased the transcription of *LDLR* gene in a fashion similar to that of MK-2206. However, similar to triciribine, CCT128930 additionally exerts a stabilizing effect on *LDLR* mRNA.

### Induction of *LDLR* gene transcription by AKT inhibitors is not secondary to ER stress

**Fig 4:**
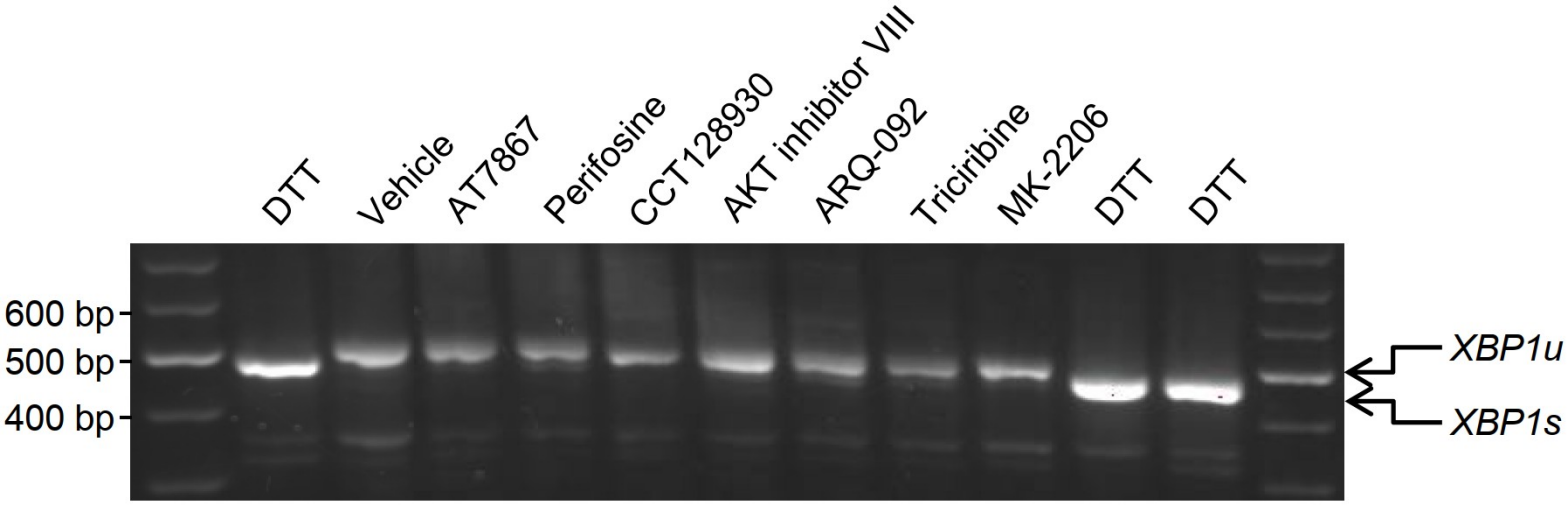
Effect of AKT inhibitors on induction of ER stress. CHO cells were treated with vehicle or the indicated AKT inhibitors as in Fig 1A. Cells treated with 5 mM DTT for 14 h served as positive control for *XBP1* mRNA splicing. Total RNA was isolated, subjected to RT-PCR and the RT-PCR products were resolved by agarose gel electrophoresis to separate unspliced *XBP1 (XBP1u)* and spliced *XBP1 (XBP1s)* mRNAs. One representative experiment out of three independent experiments is shown.

Transcription of the *LDLR* gene is dependent on SREBP-2, a transcription factor whose proteolytic activation is sensitive not only to intracellular cholesterol levels but also to ER stress [31, 32]. Therefore, given the observation that the majority of the AKT inhibitors used in this study stimulated the *LDLR* promoter activity (Fig 2B), we felt it important to ascertain whether the AKT inhibitors induced ER stress. To this end, we treated CHO cells with the AKT inhibitors for 14 h and examined the splicing of *XBP1* mRNA. In response to ER stress, endonuclease inositol-requiring enzyme 1-mediated unconventional splicing of *XBP1* mRNA (*XBP1u*) leads to generation of the shorter, spliced *XBP1* mRNA (*XBP1s*) [33]. As shown in Fig 4, whereas DTT, a disulfide bond reducer and an inducer of ER stress, generates *XBP1s*, none of the AKT inhibitors examined induced the expression of *XBP1s*. This result indicates that induction of *LDLR* mRNA levels by the AKT inhibitors does not occur as a consequence of ER stress.

### AKT knockdown increases *LDLR* mRNA and LDLR protein levels

All the AKT inhibitors included in this study increased the expression LDLR. However, kinase inhibitors are known to have pleiotropic effects [34]. Thus, these inhibitors could in theory mediate their effects on LDLR by mechanisms that do not involve AKT. Therefore, we decided to study the role of AKT inhibition on *LDLR* mRNA levels using siRNA-mediated gene silencing as siRNAs have been shown to exhibit high target specificity [35]. Of the three AKT isoforms, AKT1 is ubiquitously expressed, while AKT2 is primarily expressed in insulin-responsive tissues [36]. Because AKT3 is mainly expressed in testes and the brain and is nearly undetectable in liver-derived cells [36], we focused on the other two AKT isoforms.

**Fig 5:**
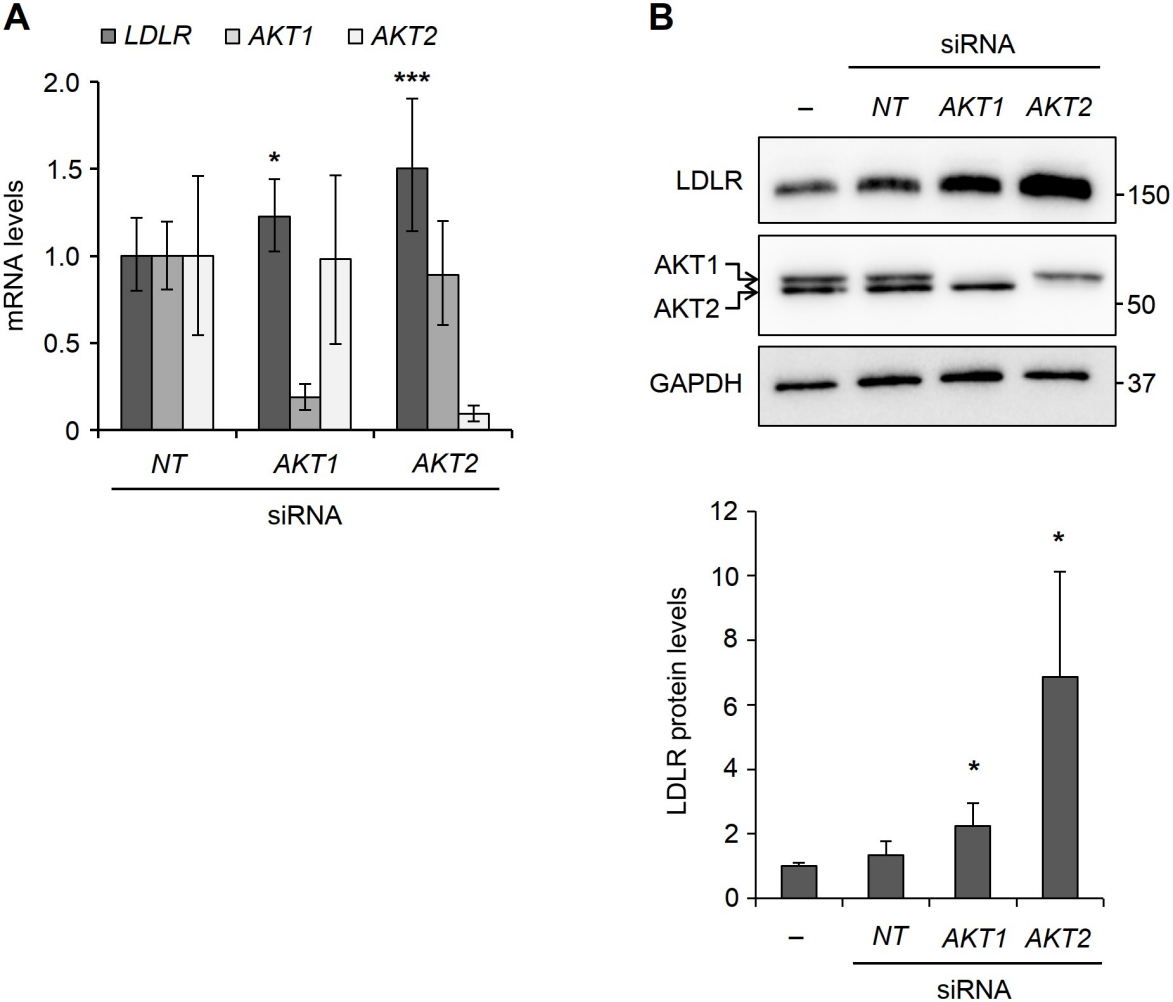
Effect of AKT isoform-specific knockdown on LDLR levels. (A) HepG2 cells were transfected with non-targeting (*NT*) siRNA, *AKT1* siRNA or *AKT2* siRNA. At 40 h post-transfection, cells were harvested for determination of *LDLR*, *AKT1*, *AKT2* and *GAPDH* mRNA levels by qPCR. After normalization to *GAPDH*, *LDLR*,*AKT1* and *AKT2* mRNA levels were plotted relative to those obtained for vehicle-treated, *NT* siRNA-transfected cells. Error bars represent the 95% confidence interval. *p < 0.05, **p < 0.01 and ***p < 0.001 relative to matched, vehicle-treated, NT siRNA-transfected cells.(B) HepG2 cells were transfected and harvested as in A and then processed for determination of LDLR and AKT protein levels by Western blotting with the indicated antibodies. One representative blot of eight is shown. Error bars represent SD. *p < 0.05 relative to matched, vehicle-treated, NT siRNA-transfected cells.

For these studies, HepG2 cells were transfected with siRNAs against *AKT1* or *AKT2*. Transfected cells were cultured for 40 h and the level of *LDLR* mRNA was determined by qPCR. As can be seen from Fig 5A, *AKT1* and *AKT2* mRNA levels were reduced by 81% and 90%, respectively, in siRNA-treated cells. Importantly, knockdown of AKT1 or AKT2 led to induction of *LDLR* mRNA levels by 23% and 50%, respectively (Fig 5A).

To examine whether knockdown of AKT1 or AKT2 also increased LDLR protein levels, HepG2 cells were transfected with siRNAs against *AKT1* or *AKT2* and LDLR protein levels was determined by Western blot analysis. As can be seen from Fig 5B, knockdown of AKT1 or AKT2 increased the LDLR protein level, suggesting that the effect of the AKT inhibitors on LDLR is mediated by their inhibitory effect on AKT.

### AKT1 and AK2 affect *LDLR* mRNA levels by different mechanisms

**Fig 6:**
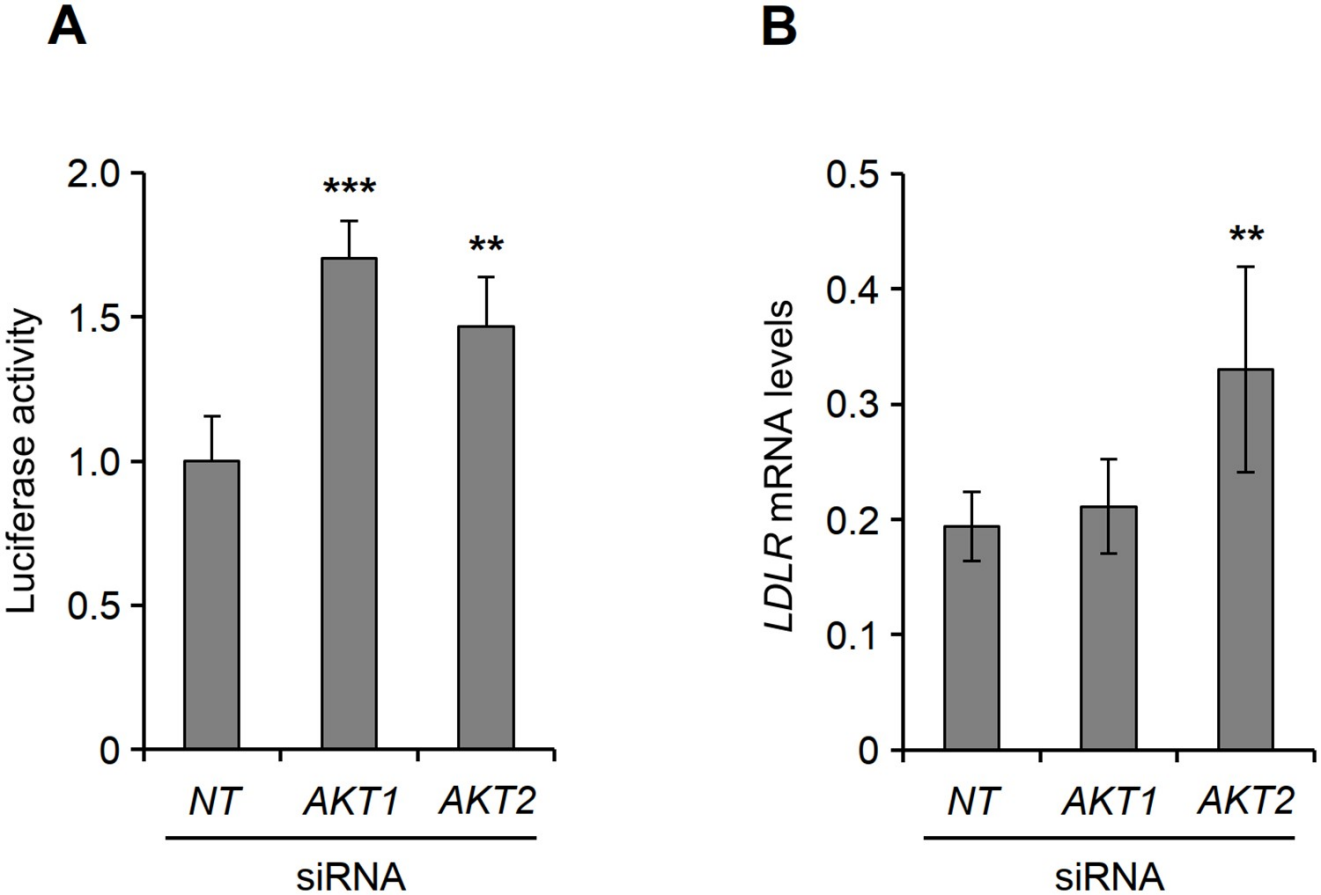
Effect of AKT isoform-specific knockdown on *LDLR* gene promoter activity and mRNA stability. (A) HepG2 cells were co-transfected with the luciferase reporter plasmid pLR1563-luc and non-targeting (*NT*), *AKT1* or *AKT2* siRNA, and then harvested after 40 h for measurement of luciferase activity. The value obtained from cells transfected with *NT* siRNA was set as 1. The graph shows the mean (±SD) values from four independent experiments. **p < 0.01, ***p < 0.001 relative to cells transfected with *NT* siRNA. (B) HepG2 cells were transfected with *NT* siRNA, *AKT1* siRNA or *AKT2* siRNA. After 40 h, 5 *μ*g/ml Act D was added and cells were then harvested at 0 and 6 h for measurement of *LDLR* and *GAPDH* mRNA levels by qPCR. *LDLR* mRNA levels were normalized to those of GAPDH, and the amount of *LDLR* mRNA were normalized relative to its internal 0-hour control sample, which was set as 1. The graph shows the 6-hour samples with the mean (±confidential interval) values calculated from four independent experiments. **p < 0.01 relative to the amount of *LDLR* mRNA before the addition of Act D.

Assuming that the kinase inhibitors used in this study exhibit AKT isoform preference *in vivo*, the dichotomy between the mechanisms that are utilized by different AKT inhibitors to induce the expression of LDLR would suggest that AKT regulates the LDLR expression machinery in an isoform-specific manner. To assess the plausibility of this notion, we decided to examine the effect of AKT isoform-specific knockdown on *LDLR* promoter activity and *LDLR* mRNA stability. First, we examined the luciferase activity in HepG2 cells that were co-transfected with the *LDLR* promoter reporter plasmid pLR1563-luc and either *AKT1*-specific siRNA or *AKT2*-specific siRNA. As shown in Fig 6A, treatment of cells with either *AKT1* siRNA or *AKT2* siRNA led to induction of luciferase activity by 70% and 47%, respectively. To examine whether AKT-specific knockdown affects *LDLR* mRNA stability, we cultured HepG2 cells that were transfected with *AKT1* or *AKT2* siRNAs in the absence or presence of Act D for 6 h and then examined *LDLR* mRNA levels by qPCR. As shown in Fig 6B, only AKT2 knockdown resulted in increased *LDLR* mRNA stability. Taken together, these results suggest that while both AKT1 and AKT2 are involved in regulation of LDLR expression, only AKT2 plays a role in modulation of *LDLR* mRNA stability.

## Discussion

We have recently shown that inhibition of AKT by two small molecular inhibitors, MK-2206 and triciribine, increases the level of *LDLR* mRNA [16, 17]. Mechanistically, we showed that MK-2206 induces the proteolytic cleavage of SREBP-2 and thus stimulates *LDLR* gene transcription. In contrast, triciribine induces *LDLR* mRNA levels by decreasing its turnover rate. Given the potential for off-target effects often associated with pharmacological inhibition agents, we considered it necessary to verify or falsify whether this apparent discrepancy between the effects of MK-2206 and triciribine on the LDLR expression machinery occurs as the result of inhibition of kinases other than AKT. To this end, we argued that structurally and functionally different kinase inhibitors that share the same target are highly unlikely to produce the same off-target effect and initiated the current study to examine the effect of five additional AKT inhibitors on LDLR expression. This panel of AKT inhibitors consisted of two allosteric inhibitors, ARQ-092 and AKT inhibitor VIII, one PH domain-interacting inhibitor, perifosine, and two ATP-competitive inhibitors, AT78806 and CCT128930. As expected, all AKT inhibitors inhibited AKT and its downstream substrates PRAS40 and GSK3*β*, but had no effect on ERK-phosphorylation (S1 Fig). We found that, similar to MK-2206, all inhibitors used in this study exhibited an inducing effect on *LDLR* promoter activity, albeit with varying degree. In contrast, only the ATP-competitive and AKT2-specific inhibitor, CCT128930, behaved as triciribine and increased the stability of *LDLR* mRNA. These results strongly suggested that inhibition of AKT is responsible for upregulation of LDLR expression but did not shed light on the mechanism that dictates the mode of AKT inhibition-mediated induction of LDLR. Initially, we hypothesized that the inhibitor-bound conformation of AKT might determine whether LDLR expression is induced by stimulation of its promoter activity or inhibition of its mRNA degradation. If this were the case, then inhibitors that induce similar conformational changes in AKT would be expected to induce LDLR expression by the same mechanism. However, the lack of congruence between the LDLR-inducing mechanisms utilized by inhibitors with similar mode of action (S2 Fig) suggested that this hypothesis is unlikely to be true. Given the fact that AKT family consists of three isoforms that exhibit distinct, non-redundant functions [37], together with the observation that the AKT2-specific inhibitor, CCT128930, was the only inhibitor that, increased *LDLR* mRNA stability, led us to consider the possibilty that AKT isoforms might differentially regulate LDLR expression. Indeed, the result obtained using AKT isoform-specific siRNAs lend support to this hypothesis and suggest that while inhibition of AKT1 increases *LDLR* promoter activity, abrogation of AKT2 induces not only *LDLR* gene expression but also the stability of its mRNA. In support of this conclusion, we would like to note that the notion that different inhibitors of the same kinase use different mechanisms to achieve the same phenotype is not without precedence. For instance, a metaanalysis of the effect of various inhibitors of the epidermal growth factor receptor has shown that whereas Gefitinib inhibits cellular proliferation by inducing the expression of cell cycle inhibitors, Erlotinib does so by suppressing the expression of cell cycle promoters [38].

Our data showing that inhibition of AKT increases LDLR levels suggest that inhibitors of AKT may have potential as lipid-lowering drugs. However, given the involvement of AKT in a diverse set of cellular processes, it is reasonable to raise concern that AKT inhibitors might cause unacceptable side-effects that would preclude their use as hypocholesterolemic agents. Indeed, results from several cancer clinical trials concerning the anti-neoplastic effect of AKT inhibitors show that inhibition of AKT is associated with a number of side-effects [39–42]. It is therefore of considerable importance to examine whether the AKT inhibitor doses needed to achieve a sufficient reduction in LDL cholesterol levels would cause adverse effects. Should this prove to be the case, use of AKT isoform-specific inhibitors would allow for reduction of LDL cholesterol levels without inhibition of the AKT isoform whose inhibition is associated with side-effects.

## Supporting information

S1 Fig Effect of AKT inhibitors on AKT isoforms, AKT substrates and ERK. (PDF)

S2 Fig Schematic representation of the mode of action of AKT inhibitors. (PDF)
