# Supplementary material for: Inhibitors of AKT kinase increase LDL receptor mRNA expression by two different mechanisms
Source: PLoS One. 2019 Jun 19;14(6):e0218537. doi: 10.1371/journal.pone.0218537 (PMC6583949; doi:10.1371/journal.pone.0218537)
Supplement: S1 Fig — (PDF) [file pone.0218537.s001.pdf]

Inhibitors of AKT kinase increase LDL receptor mRNA expression by two different mechanisms

Katrine Bjune*, Lene Wierød and Soheil Naderi

Unit for Cardiac and Cardiovascular Genetics, Department of Medical Genetics, Oslo University Hospital, Oslo, Norway

***Corresponding authors**. Unit for Cardiac and Cardiovascular Genetics, Department of Medical Genetics, Oslo University Hospital, Oslo, Norway

e-mail: kabjun@ous-hf.no

Supplementary figures and figure legends

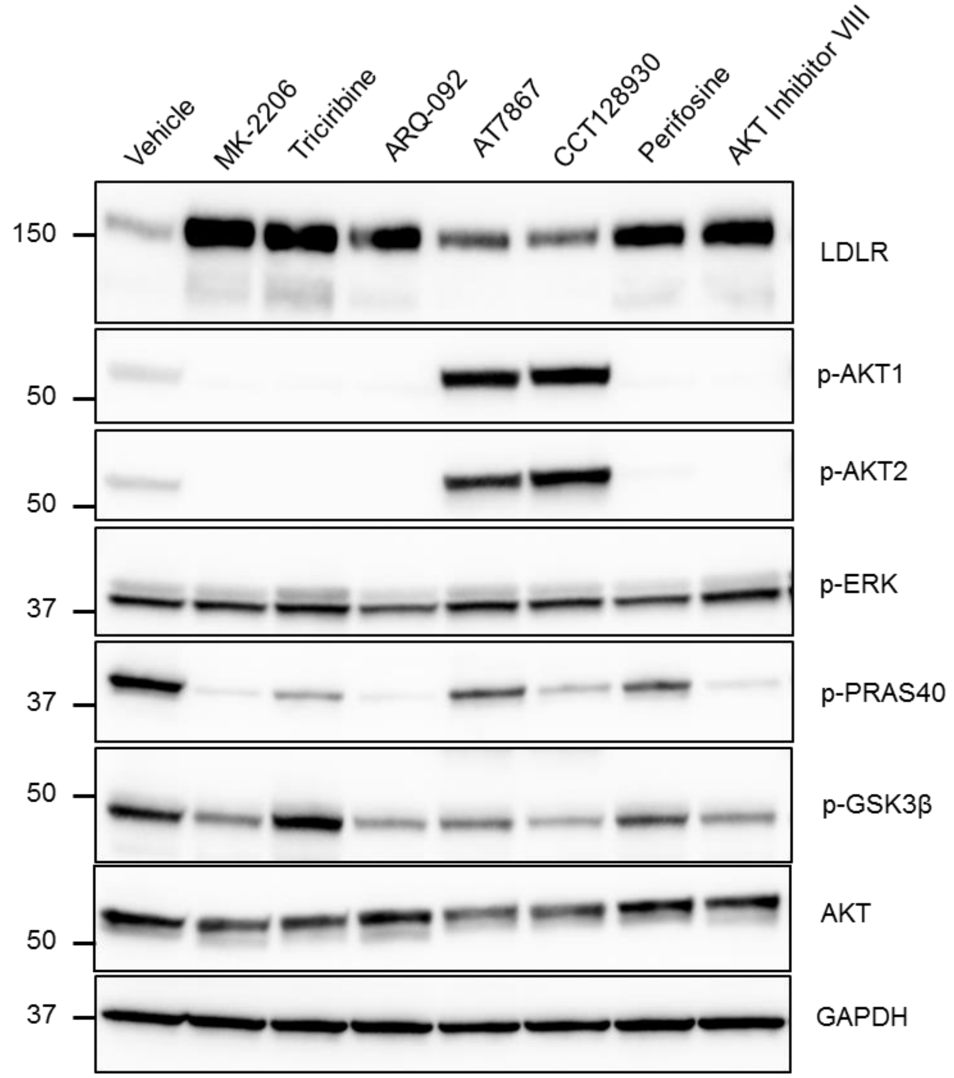

S1 Fig. Effect of AKT inhibitors on AKT isoforms, AKT substrates and ERK. HepG2 cells were treated with vehicle, 5 µM MK-2206, 1 µM triciribine, 4 µM ARQ-092, 5 µM AT7867, 10 µM CCT128939, 30 µM perifosine or 7.5 µM AKT inhibitor VIII for 8 h before harvesting for analysis by immunoblotting with antibodies against pan AKT (4691), phosphorylated AKT1 (9018), phosphorylated AKT2 (8599) and phosphorylated PRAS40 (2997) all obtained from Cell Signaling (Danvers, MA), antibodies against phosphorylated GSK3-β (AF1590) and phosphorylated ERK (AF1018) obtained from R&D systems (Minneapolis, MN) and antibody against LDLR (3839-100) purchased from BioVision (Milpitas, CA). The figure shows one representative blot from four independent experiments.
