# Supplementary material for: Inhibitors of AKT kinase increase LDL receptor mRNA expression by two different mechanisms
Source: PLoS One. 2019 Jun 19;14(6):e0218537. doi: 10.1371/journal.pone.0218537 (PMC6583949; doi:10.1371/journal.pone.0218537)
Supplement: S2 Fig — (PDF) [file pone.0218537.s002.pdf]

Inhibitors of AKT kinase increase LDL receptor mRNA expression by two different mechanisms

Katrine Bjune*, Lene Wierød and Soheil Naderi

Unit for Cardiac and Cardiovascular Genetics, Department of Medical Genetics, Oslo University Hospital, Oslo, Norway

***Corresponding authors**. Unit for Cardiac and Cardiovascular Genetics, Department of Medical Genetics, Oslo University Hospital, Oslo, Norway

e-mail: kabjun@ous-hf.no

Supplementary figures and figure legends

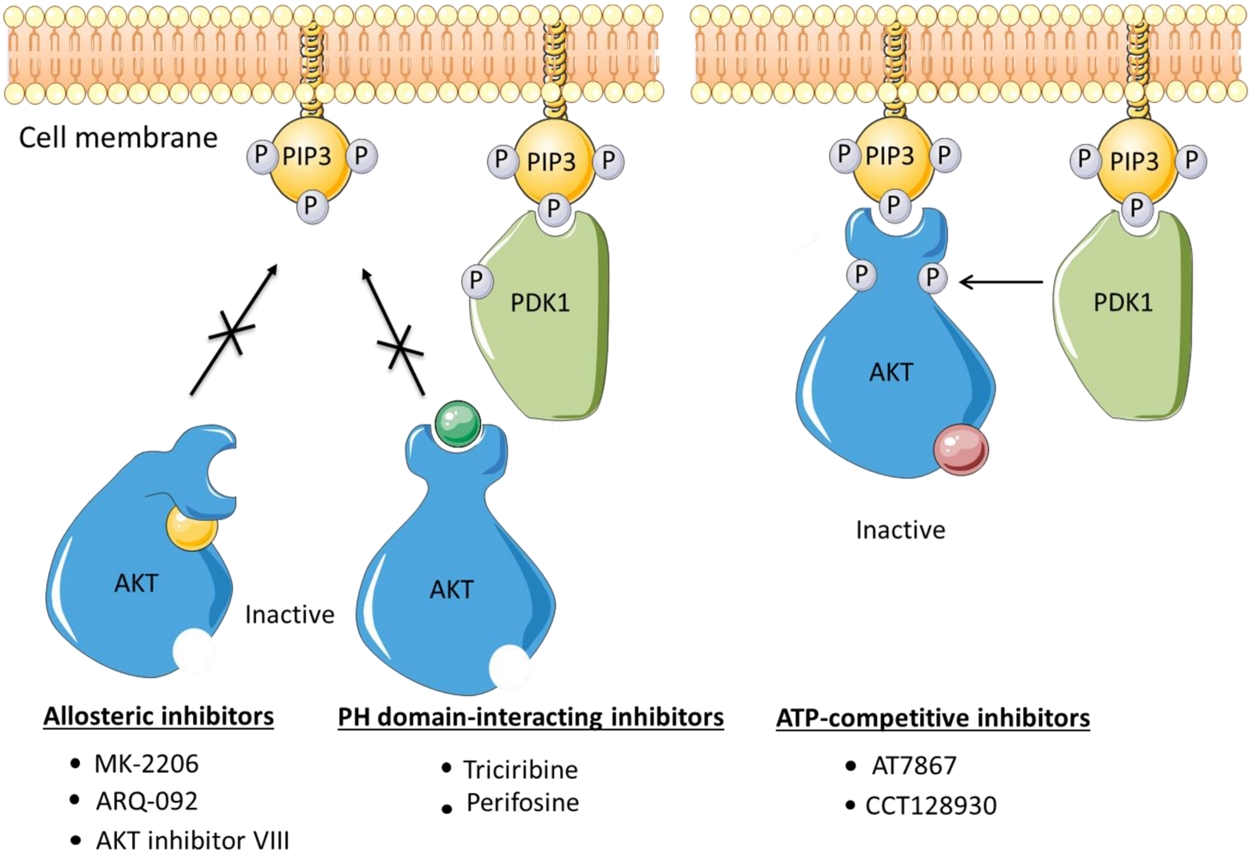

S2 Fig. Schematic representation of the mode of action of AKT inhibitors. Allosteric inhibitors bind to and lock AKT in a closed, PH-in, conformation. PH domain-interacting inhibitors prevent binding of AKT PH domain to PIP3. Both allosteric PH domain-interacting inhibitors prevent the activating phosphorylation of AKT by inhibiting its membrane translocation. ATP-competitive competitive inhibitors bind the phosphorylated, active conformation and trap AKT in an enzymatically inactive state unable to bind ATP.
